# Prognostic impact of tumor size on patients with neuroblastoma in a SEER‐based study

**DOI:** 10.1002/cam4.4653

**Published:** 2022-03-22

**Authors:** Jin‐Xia Wang, Zi‐Yang Cao, Chun‐Xia Wang, Hong‐Yang Zhang, Fei‐Long Fan, Jun Zhang, Xiao‐Yan He, Nan‐Jing Liu, Jiang‐Bin Liu, Lin Zou

**Affiliations:** ^1^ Clinical Research Unit Children's Hospital of Shanghai Jiaotong University Shanghai China; ^2^ Institute of Pediatric Infection, Immunity, and Critical Care Medicine, Shanghai Children's Hospital Shanghai Jiao Tong University School of Medicine Shanghai China; ^3^ General Surgery Department Children's Hospital of Shanghai Jiaotong University Shanghai China; ^4^ Surgical Oncology Department Children's Hospital of Chongqing Medical University Chongqing China; ^5^ Center for Clinical Molecular Medicine Children's Hospital of Chongqing Medical University Chongqing China; ^6^ Department of Clinical Laboratory, Chongqing Key Laboratory of Translational Research for Cancer Metastasis and Individualized Treatment Chongqing University Cancer Hospital & Chongqing Cancer Institute & Chongqing Cancer Hospital Chongqing China

**Keywords:** neuroblastoma, prognosis, SEER, survival analysis, tumor size

## Abstract

**Objective:**

The prognostic value of tumor size in neuroblastoma (NB) patients has not been fully evaluated. Our purpose is to elucidate the prognostic significance of tumor size in surgery performed on neuroblastoma patients.

**Methods:**

Neuroblastoma patients diagnosed from 2004 to 2015 were selected from the Surveillance, Epidemiology, and End Results Program (SEER) for the study. Univariate and multivariate Cox proportional hazard regression models were used to identify risk factors and the independent prognostic influences of tumor size on NB patients. Overall survival (OS) was analyzed through univariate Cox regression analysis. To determine the optimal cutoff value of tumor size, we first divided the cohort into three groups (≤5 cm, 5–10 cm, >10 cm). Subsequently, the patients were divided into two groups repeatedly, with tumor size at 1 cm intervals. The cutoff value that maximized prognostic outcome difference was selected. Furthermore, we performed the Kaplan–Meier methods to visually present differences in prognosis between the optimal tumor size cutoff value in different subgroups.

**Results:**

A total of 591 NB patients who met the inclusion criteria were selected from the SEER database in this study. Cox analysis showed that age >1 year (HR = 2.42, *p* < 0.0001), originate from adrenal site (HR = 1.7, *p* = 0.014), distant stage (HR = 6.4, *p* < 0.0001), undifferentiated grade (HR = 1.94, *p* = 0.002), and large tumor size (HR = 1.5, *p* < 0.0001) independently predicted poor prognosis. For tumor size, there were significant differences in tumor size distribution in different ages, tumor grade, disease stage, and primary site subgroup but not in sex, race, and histology subgroup. Furthermore, both univariate (HR = 4.96, 95% CI 2.31–10.63, *p* < 0.0001) and multivariable analysis (HR = 2.8, 95% CI 1.29–6.08, *p* < 0.0001) indicated the optimal cutoff value of tumor size was 4 cm for overall survival of NB patients. Using a 4 cm of tumor size cutoff in subgroups, we found that it can identify poor prognosis patients whatever their age or primary site. Interestingly, tumor size of 4 cm cutoff can only identify unfavorable NB patients with diagnosis at distant‐stage disease, or differentiated grade tumor, but not with regional and local or undifferentiated tumor.

**Conclusions:**

Tumor size is first to be recognized as a key prognostic factor of neuroblastoma patients and a cutoff value >4 cm might predict poor prognosis, which should be included in the evaluation of prognostic factors for NB.

## Graphical abstract

we found that tumor size could independently predict prognosis in NB patients, and a single cut‐off at 4 cm that maximized prognostic discrimination of NB children was essential for identify the poor prognosis subgroup.

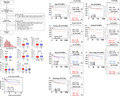

## 1 INTRODUCTION

Neuroblastoma (NB) is the most common extracranial solid tumor in pediatric and the overall incidence is 6–8 cases per million deaths,^1^ which represents about 8% of all malignant tumors diagnosed in children, and responsible for approximately 15% of all childhood cancer deaths.^2^, ^3^ The most common age of onset is between 18 and 22 months, with most patients diagnosed before the age of five.^4^ NB is characterized as heterogeneity of clinical manifestations and prognosis, of which patients with high risk have a poor prognosis, with an overall 5‐year survival rate of less than 50% compared to that with low‐risk patients.^3^ Therefore, it is critical to predict the outcome of neuroblastoma patients by appropriate risk‐stratification.

Over the last several decades, to accurately predict the outcome of neuroblastoma, several clinical and biological prognostic factors have been identified and included in the risk classification system, which helps explain the clinical behaviors of NB. The Children's Oncology Group (COG) has applied disease stage, age at diagnosis, MYCN status, tumor histology, and DNA index to stratify patients' prognostic risk.^5^ While the International Neuroblastoma Risk Group (INRG) analyzed 8800 childhood NB patients and identified seven potential prognostic factors to category patients into very low, low, intermediate, and high‐risk diseases, which including age, INRG stage, tumor differentiation grade, histologic subtypes, absence/presence of 11q aberrations, MYCN status, and tumor cell ploidy.^6^, ^7^ However, tumor size, as one of the valuable clinicopathological features, was not included in these classification systems.

Tumor size is defined as the maximum diameter of tumor and serves as a critical factor for the prognosis of solid tumors.^8^ It has been reported that tumor size is correlated with the prognosis of lung cancer,^9^ hepatocellular cancer,^10^ and gastric cancer,^11^ which indicates that tumor size is crucial for evaluating patients' prognosis. For neuroblastoma, only a few studies have evaluated the impact of tumor size on the prognosis of patients.^12^, ^13^ These studies, however, are limited to the small population of patients. Therefore, whether tumor size is an independent prognostic factor for the overall survival of neuroblastoma patients, what is the optimal cutoff value of tumor size, and whether it can identify new prognostic subgroups combined with other prognostic factors remains to be determined.

In the current study, we investigated the independent prognostic value of tumor size in surgery performed on NB patients, and determined the optimal cutoff value of tumor size to identify patients with poor prognosis. Moreover, we subdivided NB patients into a prognostic relevant subgroup by combining tumor size with other specific factors. Clarifying the impacts of tumor size on the prognosis of NB patients is helpful to determine appropriate therapeutic strategies, especially for surgical treatment.

## 2 METHODS

### 2.1 Patients

The study population was obtained from the Surveillance, Epidemiology, and End Results Program (SEER), which covers approximately 30% of the US population. Patients diagnosed with neuroblastoma (ICD‐O‐3: 9500/3, 9490/3) as a first primary malignant tumor between 2000 and 2018 were chosen for this study. Only histologically confirmed and surgery performed malignancy of the neuroblastoma were included. Moreover, to ensure the integrity and quality of clinical information, we selected those patients who were included in TNM 7/CS v0204+ Schema from 2004 to 2015. Variables in the study included sex, age at diagnosis, race, primary site, histologic subtype, SEER Combined Summary Stage (2004+), CS tumor size (2004–2015), and tumor grade. Tumor stages in SEER Combined Summary Stage (2004+) were defined as follows, distant (metastatic), regional (extend to adjacent tissue or lymph node involvement), or local (confined to the localized only). Tumor grades were defined as differentiated grades (including well‐differentiated I‐II and poorly differentiated III) and undifferentiated grades (IV).

**FIGURE 1:**
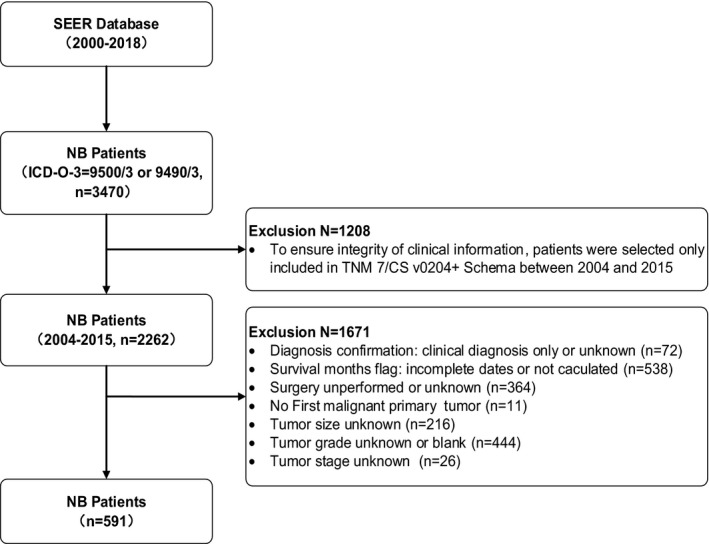
Flowchart for inclusion NB patients from SEER database

We excluded patients with incomplete survival time. Patients without cancer‐directed surgery, which surgical codes in the SEER database indicate that it was unknown or not performed cancer‐directed surgery, were excluded too. Cancers diagnosed only by death certificate or at autopsy were excluded too. Other exclusion criteria included CS tumor size code of 999 (unknown or size not stated), 990 (microscopic focus or foci only and no size of focus is given), 000 (No mass/tumor found); tumor stage unknown or unstated or unspecified, and tumor grade unknown. The procedure of the step‐by‐step extraction process of patients is presented in Figure 1, and in total, 591 patients were finally selected in the study.

### 2.2 Determine the optimal cutoff value of tumor size

To determine an optimal cutoff of tumor size, the study cohort was firstly divided into three groups, with tumor size grouped as follows: less than 5 cm, 5 to 10 cm, and larger than 10 cm, and Kaplan–Meier method was used to present prognostic differences. Then, a detail of 14 cutoffs was tested to provide an adequate level within the tumor size range by repeatedly dividing the study cohort into two groups with tumor sizes at the 1‐cm interval. Within each group, univariate, and multivariate Cox proportional hazards regression analysis of tumor size for prognostic significance was performed. For multivariate analysis, a stepwise backward model was used to identify statistically significant factors (*p* < 0.05), which adjusted for sex, age, race, histologic subtype, tumor stage, and tumor grade. In total, 17 groups were analyzed, with each divided by a different threshold of tumor size. The prognostic outcome difference between each paired group was quantified by the hazard ratio and *p*‐value. The cutoff value that maximized prognostic outcome difference, a maximum hazard rate (HR), and significant *p*‐value, between tumor size larger and smaller patients, were selected.

### 2.3 Statistical analysis

We used GraphPad Prism v7.0 (GraphPad) and SPSS v22.0 (SPSS) software for statistical analysis. Student's *t*‐test or one‐way ANOVA were used to evaluate continuous variables. To determine the independent prognostic impacts of tumor size, Cox proportional hazards regression analyses and 5‐year survival rates were performed. We used the Kaplan–Meier method to plot the survival curves between different tumor size groups (≤4.0 cm vs. >4.0 cm) in different subgroups. Hazard ratio (HR) was reported with 95% confidence interval (CI). Statistical significance was defined as *p* < 0.05.

## 3 RESULTS

### 3.1 Patients characteristics

We performed the stepwise extraction process of NB patients (Figure 1), and a total of 591 cases that met the inclusion criteria were finally selected in this study. The clinical and demographic characteristics for these patients are listed in Table 1. Among them, 59.39% of patients (𝑛 = 351) were ≤1 year old, 74.11% (𝑛 =438) were white, 55% (𝑛 = 325) were male. On histologic subtype, most patients were neuroblastoma (93.23%, 𝑛 = 551). Of tumor stage, most was diagnosed at distant stage (50.08%, 𝑛 = 296), with 28.26% at regional and 21.66% at a localized stage. At last contact, 122 patients (20.64%) died. The median follow‐up time was 69 months (IQR, 73 months), and the mean ± SD time was 77.92 ± 47.32 months. The median tumor size was 6.60 cm (IQR, 5.30 cm), and the mean ± SD was 7.26 ± 3.72 cm (Table 1).

**TABLE 1:** Characteristics of patients from SEER database

	Group	Number (*N* = 591)	Percent (%)
Age	≤1y	351	59.39
	>1y	240	40.61
Gender	Female	266	45
	Male	325	55
Race	White	438	74.11
	Black	96	16.24
	Others	57	9.65
Histology	NB	551	93.23
	GNB	40	6.77
Primary site	Adrenal Non‐adrenal	316	53.47
	Non‐adrenal	275	46.53
Tumor Grade	Differentiated	521	88.16
	Undifferentiated	70	11.84
Tumor Stage	Distant	296	50.08
	Localized	128	21.66
	Regional	167	28.26
Vital Status	Live	469	79.36
	Death	122	20.64
Tumor Size (cm)	Mean (SD)	7.26 (3.72)	–
	Median (IQR)	6.60 (5.30)	–
Follow Up (months)	Mean (SD)	77.92 (47.32)	–
	Median (IQR)	69 (73)	–

Abbreviations: 1y, 1 year old; GNB, ganglioneuroblastoma; IQR, interquartile range; NB, neuroblastoma; SD, standard deviation.

### 3.2 Univariate and multivariate Cox analysis for overall survival

Univariate Cox analysis indicated that age (HR = 4.82, *p* < 0.0001), primary site (HR = 2.62, *p* < 0.0001), tumor grade (HR = 2.86, *p* < 0.0001), distant tumor stage (HR = 9.23, *p* < 0.0001), and tumor size (HR = 2.04, *p* < 0.0001) were significantly associated with the prognosis of NB patients, whereas sex (*p* = 0.851), race (P >0.292) and histologic subtype (*p* = 0.844) were not associated (Table 2). Furthermore, multivariate Cox analysis showed that age > 1y (HR = 2.42, *p* < 0.0001), adrenal site (HR = 1.7, *p* = 0.014), undifferentiated grade (HR = 1.94, *p* = 0.002), distant stage (HR = 6.4, *p* < 0.0001), and large tumor size (HR = 1.5, *p* < 0.0001) independently predicted poor prognosis of NB patients (Table 2).

**TABLE 2:** Univariate and multivariate Cox proportional hazard regression model of overall survival

Characteristics	Group	Univariate analysis		*p*	Multivariate analysis		*p*
		HR	95% CI		HR	95% CI	
Age	>1 y	4.82	3.21–7.25	<0.0001	2.42	1.55–3.77	<0.0001
	≤1 y	ref			ref		
Gender	Female	1.04	0.72–1.48	0.851	0.76	0.53–1.10	0.147
	Male	ref			ref		
Race	White	0.73	0.41–1.31	0.292	0.56	0.31–1.02	0.06
	Black	1.29	0.67–2.48	0.441	0.65	0.33–1.27	0.207
	Others	ref			ref		
Primary site	Adrenal	2.62	1.75–3.92	<0.0001	1.70	1.11–2.59	0.014
	Non‐adrenal	ref			ref		
Histology	NB	1.07	0.54–2.11	0.844	1.06	0.52–2.14	0.878
	GNB	ref			ref		
Tumor grade	Undifferentiated	2.86	1.90–4.30	<0.0001	1.94	1.28–2.96	0.002
	Differentiated	ref			ref		
Tumor stage	Distant	9.23	4.51–18.92	<0.0001	6.40	3.07–13.34	<0.0001
	Regional	0.32	0.07–1.51	0.15	0.36	0.18–1.68	0.192
	Localized	ref			ref		
Tumor size (cm)	Mean, SD	2.04	1.68–2.48	<0.0001	1.50	1.22–1.85	<0.0001

Abbreviations: 1y,1 year old; CI, confidence interval; GNB, ganglioneuroblastomae; HR, hazard ratio; NB, neuroblastoma; ref, reference; SD, standard deviation.

### 3.3 Distribution of NB patients according to tumor size

**FIGURE 2:**
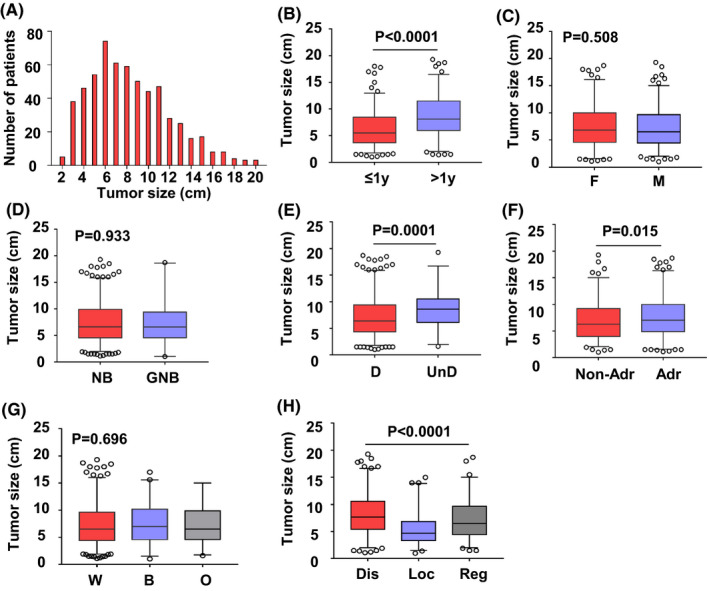
The NB patients' distribution according to tumor size. The boxes show the upper quartile, median, and lower quartile. Bars indicate the 95% CI, with lower 97.5 percentile and upper 2.5 percentile. The little circles stand for outlier observations beyond 95% CI. The lines on the top of boxes indicate a significant statistical comparison between subgroups. (A) Tumor size distribution in total population; (B–H) Comparison of the tumor size distribution between different subgroups [B, age group (y, year); (C), sex group (F, female; M, male); (D) histology group (NB, neuroblastoma; GNB, ganglioneuroblastoma); (E) tumor grade (D, differentiated; UnD, undifferentiated); (F) primary site (Non‐Adr, non‐adrenal; Adr, adrenal); (G) race group (W, white; B, black; O, others); (H) disease stage (Dis, distant; Loc, local; Reg, regional)

To further know the frequency of patient distribution by tumor size, we collected and analyzed the information as shown in Figure 2. Overall, the median tumor size in the total population was 6.60 cm, and the mean tumor size was 7.26 ± 3.72 cm (Figure 2A). For demographic characteristics, the median tumor size for children less than 1‐year‐old (5.5 cm, IQR: 3.7–8.5 cm) was significantly smaller than those older children (8.1 cm, IQR: 6.1–11.5 cm) (Figure 2B). While tumor size distribution between girls and boys (*p* = 0.508), or different races (*p* = 0.696), was not statistically significant (Figure 2C, G). For biological characteristics of the tumor, the median size for distant tumor stage (7.7 cm, IQR: 5.4–10.6 cm) was significantly larger compared with those for regional (6.5 cm, IQR: 4.4–9.7 cm) and localized (4.7 cm, IQR: 3.3–6.8 cm) stage (Figure 2H, One‐way ANOVA, *p* < 0.0001; and Tukey's multiple comparisons: distant vs. localized *p* < 0.0001; distant vs. regional *p* = 0.0042; localized vs. regional *p* < 0.0001). However, there was no significant difference was observed among patients with different histologic types (*p* = 0.933, Figure 2D). On the other hand, a significant difference was also observed between tumor differential grade (differentiated: 6.4 cm, IQR: 4.3–9.7 cm; undifferentiated: 8.6 cm, IQR: 6.1–10.6 cm; *p* < 0.005) and primary site (non‐adrenal site: 6.3 cm, IQR: 4–9.2 cm, adrenal: 7 cm, IQR: 4.9‐10 cm, *p* = 0.015), demonstrating that tumor size is closely correlated with NB's characteristics.

### 3.4 Determination of optimal tumor size cutoff for NB patients

**FIGURE 3:**
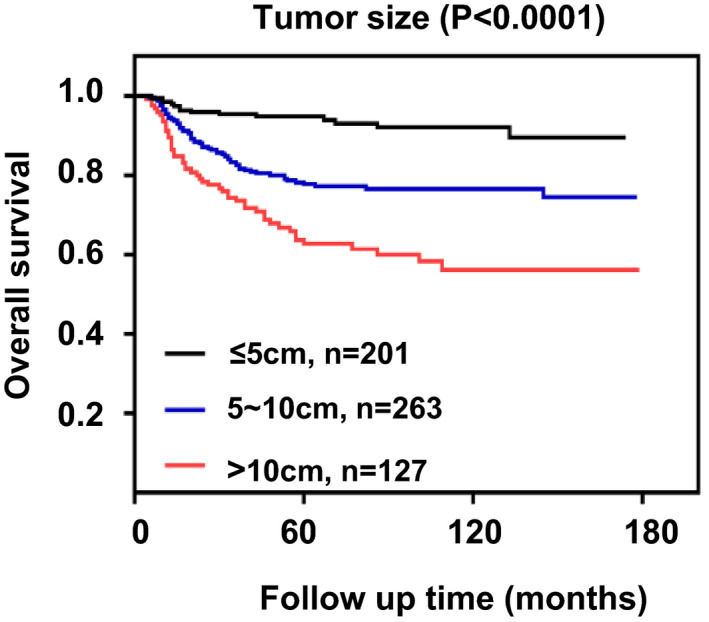
Overall survival of NB patients divided by tumor size (≤5 cm, 5–10 cm, >10 cm)

To identify a cutoff of tumor size that maximized outcome difference, 17 potential cutoffs were evaluated. First, we divided the cohort into three groups based on tumor size (≤5 cm, 5–10 cm, >10 cm), the Kaplan–Meier survival curves indicated that the survival probability of NB patients with smaller tumors was significantly higher than those with larger tumors (Figure 3). Subsequently, 14 separate analyses, each of which divided the patients into two groups, one included patient whose tumor size was larger than the cutoff value and the other one included patient whose tumor size was smaller than the cutoff value, were tested for tumor size impact on the outcome.

Univariate analysis showed that the P values of cutoff values from 3 to 15 cm were all significant and the tumor size cutoff at 4 cm had the largest HR for OS (HR = 4.96, 95% CI 2.31–10.63, *p* < 0.0001). When applying cutoff points of 4 cm, the 5‐year OS was 74% for patients with tumor size larger than 4 cm, and 94% for patients with those smaller than 4 cm (*p* < 0.001). Moreover, the optimal cut‐off remained at 4 cm after adjusted for sex, age, race, histologic subtype, tumor stage, and tumor grade (HR = 2.8, 95% CI 1.29–6.08, *p* < 0.0001), and the P values of cutoff values from 4 to 10 cm were all significant for OS. Therefore, we selected 4 cm as a stratification that optimized the prognosis of tumor size for NB children (Table 3).

**TABLE 3:** Univariate and multivariate Cox proportional hazard regression model of different tumor size cutoffs in neuroblastoma patients

Tumor size cutoff (cm)	No.	5‐years OS	Univariate analysis		*p*	Multivariate analysis		*p*
			HR (95% CI)	HR (95% CI) forest plot		HR (95% CI)	HR (95% CI) forest plot	
>2	232	75	2.47 (0.79–7.77)	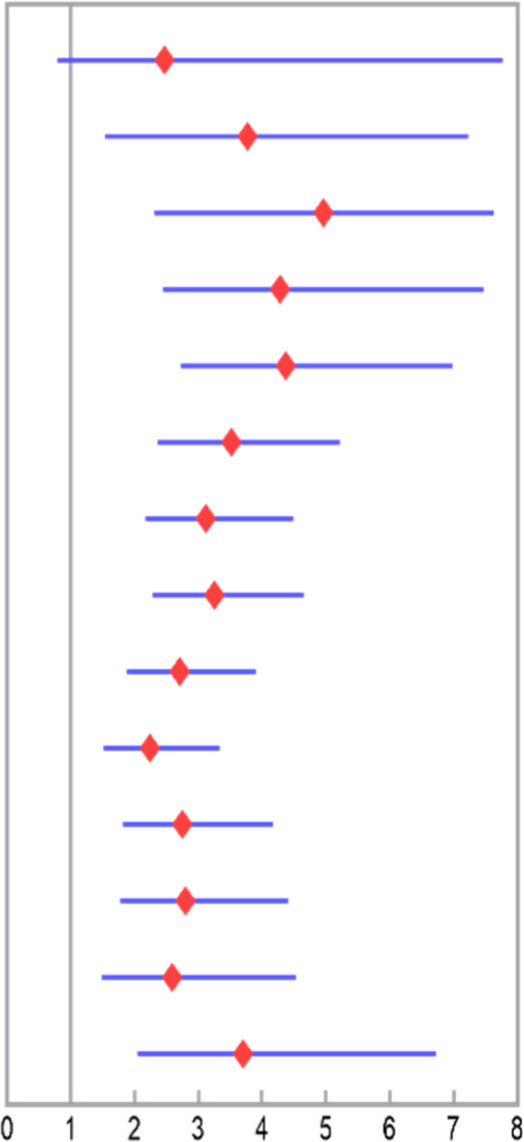	0.122	1.41 (0.45–4.48)	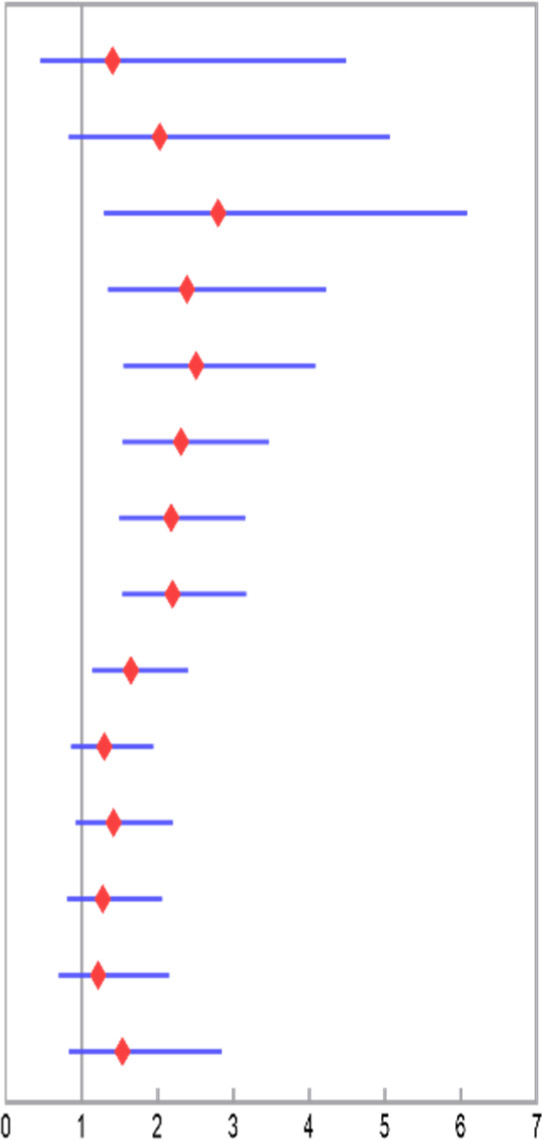	0.56
>3	224	73	3.77 (1.54–9.23)		0.004	2.03 (0.82–5.06)		0.13
>4	213	74	4.96 (2.31–10.63)		<0.0001	2.80 (1.29–6.08)		0.01
>5	195	68	4.28 (2.45–7.47)		<0.0001	2.39 (1.35–4.22)		0.003
>6	175	64	4.37 (2.73–6.99)		<0.0001	2.51 (1.55–4.08)		<0.0001
>7	147	62	3.52 (2.37–5.22)		<0.0001	2.31 (1.54–3.47)		<0.0001
>8	121	60	3.12 (2.17–4.49)		<0.0001	2.18 (1.50–3.16)		<0.0001
>9	104	56	3.25 (2.28–4.65)		<0.0001	2.20 (1.53–3.17)		<0.0001
>10	81	55	2.71 (1.88–3.90)		<0.0001	1.65 (1.14–2.4)		0.009
>11	65	56	2.24 (1.51–3.33)		<0.0001	1.30 (0.86–1.95)		0.21
>12	53	50	2.75 (1.81–4.17)		<0.0001	1.42 (0.92–2.2)		0.11
>13	41	46	2.80 (1.78–4.41)		<0.0001	1.28 (0.8–2.06)		0.31
>14	35	44	2.59 (1.48–4.52)		0.001	1.22 (0.69–2.15)		0.50
>15	16	30	3.70 (2.04–6.72)		<0.0001	1.54 (0.83–2.85)		0.17

Abbreviations: CI, confidence interval; HR, hazard ratio; No., number; OS, overall survival.

### 3.5 Impact of tumor size on overall survival in subgroups for NB patients

**FIGURE 4:**
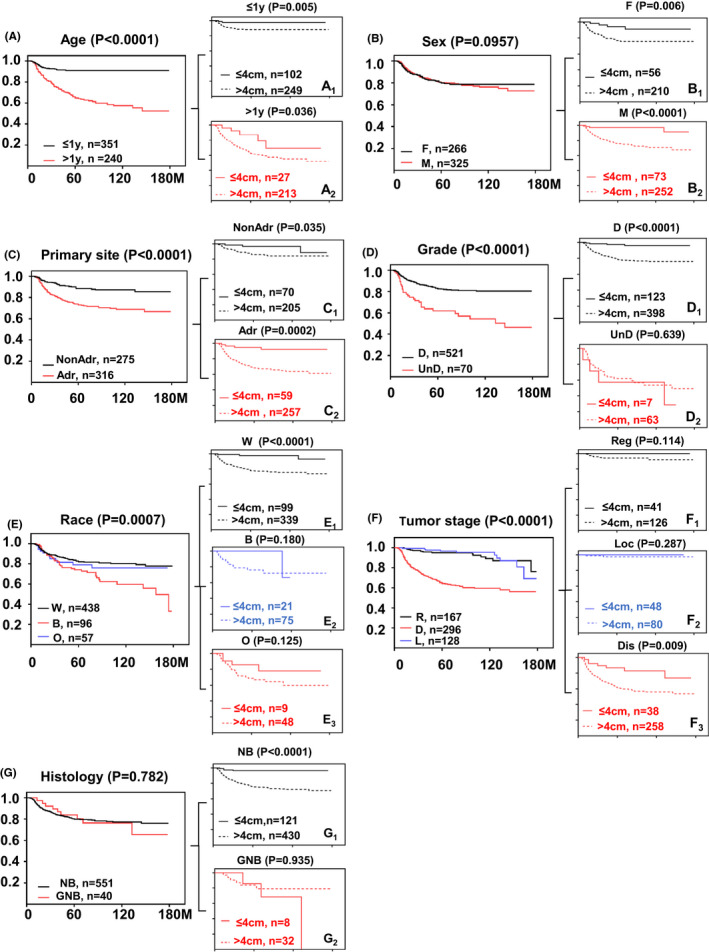
Overall survival (OS) of patient subgroups. The ordinate represents the probability of OS and the abscissa represents survival months. For each subgroup, the left figure shows the OS, and the right figure shows the subgroup survival divided by tumor size cutoff 4 cm (solid lines stand for tumor size ≤4 cm, dotted lines stand for tumor size >4 cm). (A) The OS of age subgroup (A_1_: patients≤1y; A_2_: patients >1y); (B) The OS of sex subgroup (B_1_: F, female; B_2_: M, male); (C) The OS of primary site subgroup (C_1_: Non‐Adr, non‐adrenal; C_2_: Adr, adrenal site); (D) The OS of tumor grade subgroup (D_1_: D, differentiated; D_2_: UnD, undifferentiated); (E) The OS of race subgroup (E_1_: W, white; E_2_: B, black; E_3_: O, others); (F) The OS of tumor stage subgroup (F_1_: Reg, regional; F_2_: Loc, local; F_3_: Dis, distant); (G) The OS of tumor histology subgroup (G_1_: NB, neuroblastoma; G_2_: GNB, ganglioneuroblastoma)

According to the 4 cm cutoff, the cohort was divided into two groups, with 213 (36.04%) having larger size tumors (>4 cm) and 378 (63.96%) having smaller size tumors (≤4 cm). To illustrate the interaction between other risk factors and tumor size, we then performed subgroup analysis. Survival curves stratified by the cutoff value of >4 cm presented significant differences in both age groups (<1 year, *p* = 0.005; >1 year, *p* = 0.036) (Figure 4A) and primary sites (adrenal, *p* = 0.002; non‐adrenal, *p* = 0.035) (Figure 4C). But in the race subgroup, compared to black, white patients had a favorable prognosis, in which tumor size of 4 cm was a significant predictor for OS, but not in black patients (Figure 4E). For the tumor stage subgroup, our data indicated that the overall survival of patients with large tumors at the distant stage was significantly lower than that of the small tumor group (*p* < 0.0001), but there was no significant difference in the survival rate of patients with the regional or local stage (*p* = 0.287, *p* = 0.114) (Figure 4F). For the tumor grade subgroup, survival curves showed significant differences in differentiated grade (*p* < 0.0001) tumors but not in undifferentiated grade (*p* = 0.639) (Figure 4D).

## 4 DISCUSSIONS

Neuroblastoma is known for its wide spectrum of clinical behavior, and combinations of prognostic factors are used to stratify the risk level and treatment regimen of NB patients.^6^, ^14^, ^15^ However, this classification system does not include tumor size. In the current study, we clarified that tumor size was useful in predicting the prognosis of neuroblastoma patients. Moreover, to our knowledge, this is the first time to identify a single cutoff at 4 cm that maximized prognostic discrimination. Furthermore, unfavorable prognostic subgroups of patients were identified by combining the single size threshold of 4 cm with other specific factors. These findings indicate that tumor size has a significant prognostic impact on NB patients, which provides a reference for patients' risk stratification.

Due to the clinical heterogeneity of neuroblastoma, risk assessment is clinically performed to stratify patients. The COG has applied disease stage, age at diagnosis, MYCN status, tumor histology, and DNA index to stratify patients' prognostic risk.^5^ However, tumor histology used in COG risk classification was based on International Neuroblastoma Pathology Classification (INPC) criteria, which also included age.^16^ This may result in overestimating the prognostic value of age to define risk level.^17^ While the INRG identified 7 potential prognostic factors, including age, INRG stage, tumor differentiation grade, histologic subtypes, absence/presence of 11q aberrations, MYCN status, and tumor cell ploidy,^6^, ^7^ and emphasized pretreatment risk stratification based on the clinical criteria. Our analysis suggests that age, differentiation grade, and disease stage can independently predict the prognosis of neuroblastoma, consisted of the COG and INRG risk classification system. Moreover, we found that in contrast to originate from the nonadrenal site, patients with NB originate from the primary adrenal site had poor OS, this consistent with the INRG research, that patients diagnosed at the primary adrenal site of neuroblastoma had inferior OS.^7^ Intestinally, our study indicated that white people seem to have a better prognosis than black people, which may be due to genetic differences.^4^, ^18^

The prognosis of tumor size in other solid tumors was discussed. For example, Horn et al identified a cutoff value of 2 cm of tumor size as a prognostic value in cervical cancer and suggested that tumor size ≤2.0 cm representing a low risk.^19^ Wang et al identified a tumor size threshold of 4.8 cm in gastric cancer patients,^20^ which indicates that the cutoff value of tumor size is critical for evaluating the tumor's prognosis. However, the prognostic value of tumor size for neuroblastoma patients remains unclear. Recently, a few studies have evaluated the significance of tumor size in predicting the prognosis of neuroblastoma patients. Liang et al divided patients into four subgroups based on tumor size (<5, 5.1–10, 10.1–15, and >15 cm), and found that patients with larger tumors often predict poor prognosis.^21^ Moreover, He et al found that tumor size >10 cm was related to the poor overall survival in neuroblastoma patients with bone metastasis.^22^ In contrast, other studies revealed that in univariate analysis, tumor size was a predictor of event‐free survival, but not in multivariate analysis.^13^ Although some previous studies showed that patients less than 6 months old with localized tumors <3.1 cm are at low risk and need regular observation and follow‐up,^12^ here, we identified a single cut‐off at 4 cm that might maximized prognostic discrimination in 591 patients with neuroblastoma, a relatively large sample size with 15 years' follow‐up.

However, the INRG project proposed a new staging system dependent on the presence or not the presence of image‐defined risk factors (IDRFs) in the year of 2009.^23^ A localized tumor without any IDRFs is stage L1, while a local‐regional tumor with any IDRFs is considered to be stage L2. Previous studies indicated IDRFs combination with tumor size affect prognostic outcome.^24^ Yoneda A et al found that 27% of the IDRFs became negative after chemotherapy, but for negative IDRFs, tumors should shrink to less than 20% of the volume at the time of diagnosis.^25^ In clinical practice, we found that patients with L1 stage had a good prognosis after surgical treatment. While an increasing number of IDRFs was associated with larger tumor and higher risk of NB patients in L2 stage.^26^, ^27^ Thus, a tumor size of >4 cm with L1 lesion may do not affect the outcome, but a large L2 tumor may lead to a poor prognosis. Unfortunately, the image information of neuroblastoma was not collected in the SEER database. Therefore, we cannot get the information of L1 and L2 tumors based on this database. In the future, the relationship of tumor size with IDRFs on survival needs to be further analyzed. Moreover, histology is very important to NB patients' prognosis too. Patients with favorable histology have a high survival rate, which can be greater than 90%, while those with unfavorable histology have a survival rate less than 40%,^14^ Therefore, tumor size in combination with histology types impacts on survival needs to be further analyzed in the future.

The current study has several limitations. First, due to the retrospective design, some cases were removed for incomplete data information. Secondly, patients with larger tumor size combination with image‐defined risk factors and unfavorable histology types may receive more aggressive treatment, but detailed information of the treatment, IDRFs, and histology were not included in the SEER database, hence this study may overestimate the true extent of clinical impact from increasing tumor size. To validate our research, we intended to conduct a validation study using other public databases, such as Target, but we found that the Target database lacked the variable of tumor size. Moreover, based on our hospital data, we found that tumor size was not fully documented. This may be because some researchers have not paid special attention to the significant impact of tumor size on prognosis and there is no uniform standard for determining the size of NB. Thirdly, despite being a population‐based database, after stratifying by tumor size, individual subgroups became small, this may slightly affect statistical power.

For perspective, we recommend that more attention should be paid to tumor size as a prognostic biomarker and add tumor size into the routine tumor database. Tumor size combined with other specific factors will help surgeons to better stratify and manage patients. It is possible that NB patients can be further subdivided into prognosis‐relevant subgroups by tumor size. The mechanism that may influence the occurrence and development of tumor size is worth further exploration. Prospective studies and long‐time follow‐up are necessary to further research.

In conclusion, this study first determined that tumor size is a crucial prognostic factor in neuroblastoma patients and a cutoff value >4 cm might predict a poor prognosis. Therefore, we suggest incorporating the tumor size into the risk classification system to enhance the accuracy of neuroblastoma prognostic prediction.

## CONFLICT OF INTEREST

The authors declare that they have no competing interests.

## AUTHOR CONTRIBUTIONS

Lin‐Zou and Jiang‐Bin Liu contributed to the study's conception and design. Jin‐Xia Wang analyzed the data and wrote the manuscript. Lin‐Zou and Jin‐Xia Wang revised the manuscript. Zi‐Yang Cao, Chun‐Xia Wang, Hong‐Yang Zhang, Fei‐long Fan, Xiao‐Yan He, and Nan‐Jing Liu contributed to the collection and assembly of data. Jiang‐Bin Liu and Jun Zhang provided important contributions in the field of pediatric surgery.

## ETHICAL APPROVAL STATEMENT

Because the SEER database is publicly accessible worldwide, therefore, we did not provide the approval of an institutional review board in the current study. But the authors signed the SEER database agreement and got the license to access SEER information (accession username: 12284‐Nov2020).

## Data Availability

The data is available in the SEER program
